# Cyclic *versus* Hemi-Bastadins. Pleiotropic Anti-Cancer Effects: from Apoptosis to Anti-Angiogenic and Anti-Migratory Effects

**DOI:** 10.3390/molecules18033543

**Published:** 2013-03-19

**Authors:** Véronique Mathieu, Nathalie Wauthoz, Florence Lefranc, Hendrik Niemann, Karim Amighi, Robert Kiss, Peter Proksch

**Affiliations:** 1 Laboratoire de Toxicologie, Faculté de Pharmacie, Université Libre de Bruxelles (ULB), Campus de la Plaine, Boulevard du Triomphe, 1050 Brussels, Belgium; E-Mail: rkiss@ulb.ac.be; 2 Laboratoire de Pharmacie Galénique et de Biopharmacie, Faculté de Pharmacie, Université Libre de Bruxelles (ULB), Campus de la Plaine, Boulevard du Triomphe, 1050 Brussels, Belgium; E-Mails: nawautho@ulb.ac.be (N.W.); kamighi@ulb.ac.be (K.A.); 3 Service de Neurochirurgie, Hôpital Erasme, ULB, Route de Lennik, 1070 Brussels, Belgium; E-Mail: fllefran@ulb.ac.be; 4 Institute of Pharmaceutical Biology and Biotechnology, Heinrich-Heine University Düsseldorf, Universitätsstrasse 1, 40225 Düsseldorf, Germany; E-Mails: hendrik.niemann@uni-duesseldorf.de (H.N.); proksch@uni-duesseldorf.de (P.P.)

**Keywords:** bastadins, hemibastadins, angiogenesis, apoptosis, cancer

## Abstract

Bastadins-6, -9 and -16 isolated from the marine sponge *Ianthella basta* displayed *in vitro* cytostatic and/or cytotoxic effects in six human and mouse cancer cell lines. The *in vitro* growth inhibitory effects of these bastadins were similar in cancer cell lines sensitive to pro-apoptotic stimuli *versus* cancer cell lines displaying various levels of resistance to pro-apoptotic stimuli. While about ten times less toxic than the natural cyclic bastadins, the synthetically derived 5,5'-dibromohemibastadin-1 (DBHB) displayed not only *in vitro* growth inhibitory activity in cancer cells but also anti-angiogenic properties. At a concentration of one tenth of its *in vitro* growth inhibitory concentration, DBHB displayed actual antimigratory effects in mouse B16F10 melanoma cells without any sign of cytotoxicity and/or growth inhibition. The serum concentration used in the cell culture media markedly influenced the DBHB-induced antimigratory effects in the B16F10 melanoma cell population. We are currently developing a specific inhalation formulation for DBHB enabling this compound to avoid plasmatic albumin binding through its direct delivery to the lungs to combat primary as well as secondary (metastases) tumors.

## 1. Introduction

**Figure 1:**
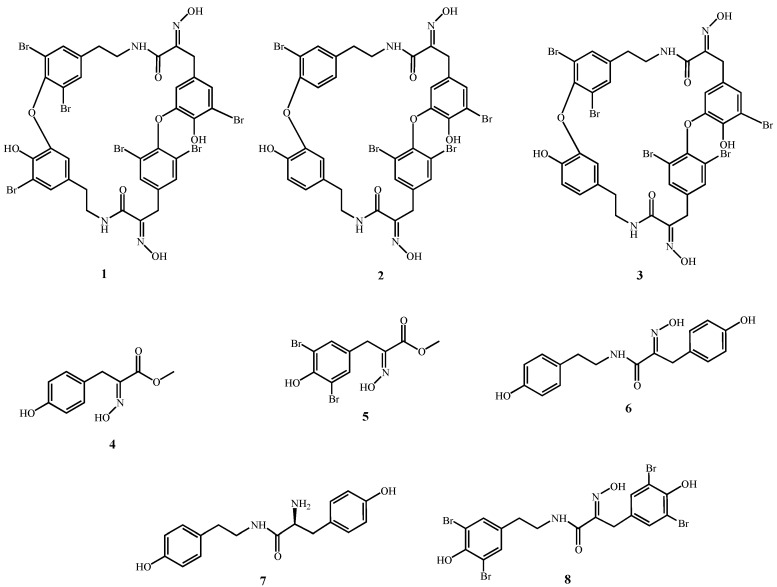
Compounds under study.

First isolated in the 1980s [1,2] and in the 1990s [3] from the sponge *Ianthella basta*, bastadins have attracted wide attention due to their pronounced biological activities. Chemically, bastadins are formed by brominated tyrosine and tyramine derivatives (Figure 1) that are linked by a peptide bond to build a hemibastadin unit. Two moieties of these putative precursors form the bastadins either by carbon bonds or by the more common ether bridges. Both, linear and macrocyclic derivatives occur in nature. The amino group of the bromotyrosine unit is typically oxidized to yield an oxime function. Approximately thirty natural bastadins have been reported so far [4,5,6,7], with full chemical syntheses successfully developed for some of them. Several novel derivatives, including hemibastadin congeners [8,9,10] that exhibit remarkable anti-fouling activity [11,12], have also been generated. For example, 5,5'-dibromohemibastadin-1 (DBHB; **8**; Figure 1) suppresses the settling of barnacle larvae through the inhibition of the blue mussel phenoloxidase that is involved in the firm attachment of fouling organisms to a given substrate [12]. Bastadins and congeners also display activity with respect to ryanodine-sensitive Ca^2+^ channels (ryanodine receptors) [13,14,15].

Several reports have described the cytotoxic activity of various bastadins towards cancer cells [16,17], as well as their anti-angiogenic activity [18,19,20]. While their pro-apoptotic effects have been demonstrated in endothelial cells with respect to their cytotoxic activity [21], the mechanisms of action through which bastadins delay cancer cell growth have not yet been elucidated, to the best of our knowledge. In addition, preliminary investigations carried out in our group showed that bastadins display similar *in vitro* growth inhibitory effects in cancer cells that display actual sensitivity to pro-apoptotic stimuli *versus* cancer cells that display various levels of resistance to pro-apoptotic stimuli (unpublished data), as it is detailed in the current study for bastadins-6 (**1**; Figure 1), -9 (**2**; Figure 1) and -16 (**3**; Figure 1), and also for DBHB (**8**; Figure 1) and other related compounds (Figure 1).

The fact that various bastadins and DBHB are able to overcome the intrinsic resistance of cancer cells to pro-apoptotic stimuli is of potential clinical importance. In addition to the well-known multidrug resistance (MDR) phenotype of various cancer cell types that resist conventional chemotherapy [22], the intrinsic resistance of cancer cells to pro-apoptotic stimuli can also lead to dismal prognoses, as reported for gliomas [23], melanomas [24], non-small-cell lung cancers (NSCLCs) [25] and esophageal cancers [26]. Metastatic cancer cells are also resistant to pro-apoptotic stimuli because they must resist anoikis during their metastatic journey [27,28]. Because a cell cannot migrate and divide simultaneously, there should be an inverse relationship between the levels of cancer cell migration and their sensitivity to pro-apoptotic stimuli [29,30]. In other words, antimigratory compounds that are not inherently cytotoxic can be as effective as cytotoxic compounds in combatting aggressive cancer cells. In addition, antimigratory compounds can increase the efficiency of cytotoxic drugs against apoptosis-resistant cancer cells, by decreasing the migration of these cancer cells [29,30]. For example, cilengitide is a cyclo[Arg-Gly-Asp-D-Phe-(NMeVal)] (cRGD) compound that acts as an antimigratory agent that targets the α(v)β(3) and α(v)β(5) integrins, which govern not only endothelial but also cancer cell adhesion; affecting thus both endothelial (angiogenesis) and cancer cell migratory (metastasis) processes, this compound has been assayed in multiple clinical trials, including studies on aggressive types of cancers [31,32].

The present study examined: (i) the characterization of the *in vitro* cytostatic *versus* cytotoxic effects of bastadins-6, -9 and -16 in multiple cancer cell lines (including several cancer cell lines displaying various levels of resistance to pro-apoptotic stimuli); (ii) the bastadin-9-induced effects on cell cycle kinetics and apoptotic features in human SKMEL-28 melanoma and U373 glioblastoma cells; (iii) the anti-angiogenic effects of DBHB; (iv) the antimigratory effects of DBHB; (v) the influence of the serum concentration in cell culture media on DBHB-induced antimigratory effects in B16F10 melanoma cells and the binding affinity of DBHB to albumin; and (vi) a first evaluation of the *in vivo* analysis of DBHB-related activity as measured by the survival of B16F10 melanoma-bearing mice.

## 2. Results and Discussion

### 2.1. *In Vitro* Growth Inhibitory Concentrations

The eight compounds whose chemical structures are illustrated in Figure 1 were assayed using the MTT colorimetric test to determine the concentration that reduced global cancer cell growth by 50% for six cancer cell lines cultured for three days in the presence of the drug of interest (Table 1). 

**Table 1:** *In vitro* growth inhibitory concentrations that reduce cell growth by 50% (IC_50_; µM) for compounds **1**–**8** (Figure 1) following culturing of the cancer cell lines with the compound of interest for 72 h (MTT colorimetric assay).

Compounds	Carcinoma		Glioma		Melanoma		Mean ± SEM
	MCF-7 (breast)	A549 (NSCLC)	Hs683	U373	B16F10 *	SKMEL28	
			(oligodendroglioma)	(astroglioma)			
1	4	3	3	3	4	4	4.0 ± 0.2
2	8	7	4	7	5	7	6.0 ± 0.6
3	7	8	4	11	6	7	7.0 ± 0.9
4	>100	>100	>100	>100	75	>100	>96
5	94	>100	>100	>100	86	>100	>97
6	>100	>100	>100	>100	45	>100	>91
7	>100	>100	>100	>100	63	>100	>94
8	68	68	70	73	58	76	69 ± 3

* All the cell lines are of human origin except the B16F10 melanoma, which is of murine origin. NSCLC means non-small-cell lung cancer.

The data shown in Table 1 clearly indicate that cyclic bastadins [bastadin-6, -9 and -16 (**1**–**3**)] display higher *in vitro* growth inhibitory effects than hemibastadins such as DBHB. However, this latter revealed a weak activity with a mean IC_50_ growth inhibitory activity of 69 µM over all cancer cell lines, including the A549 NSCLC [33], SKMEL-28 melanoma [34] and U373 [35] cell lines that exhibit various levels of resistance to pro-apoptotic stimuli. The cancer cell lines sensitive to pro-apoptotic stimuli, including the MCF-7 breast cancer [36], the Hs683 oligodendroglioma [35] and the B16F10 melanoma [34] cell lines, did not display higher sensitivity to bastadins and DBHB than the A549, SKMEL-28 and U373 cancer cells. These data suggest that bastadins and DBHB display their anti-cancer activities regardless to their sensitivity to apoptosis. Therefore, we hypothesized that induction of apoptosis should not be the primary mechanism of action of these compounds that could thus be used to combat models associated with, at least partial, intrinsic resistance to pro-apoptotic stimuli. If at first glance, DBHB appeared to have rather weak activity (at least in terms of IC_50_ *in vitro* growth inhibitory concentrations), it must kept in mind that temozolomide, the most efficacious drug used clinically to treat glioblastoma [23], has IC_50_ *in vitro* growth inhibitory concentrations ranging between 220 (U373) and 956 (Hs683) µM, depending on the glioma cell line [37]. The same features were observed for another widely used compound to combat various cancer types, *i.e.*, carboplatin, whose IC_50_ growth inhibitory concentrations ranged between 11 (A549) and 149 (SKMEL-28) µM [38].

A comparison of the analyzed compounds clearly reveals that the studied bastadin derivatives **1**–**3**, which can be envisioned as dimeric hemibastadin congeners, show the strongest activity in all cell lines investigated in this study. Differences in activity between the individual bastadins are only minor, as reflected by their similar IC_50_ values. Among the synthetically derived hemibastadin derivatives **6**–**8**, compound **8** (DBHB) shows the best activity, which can be traced back to its brominated aromatic rings, as is evident from comparison of **8** with the debromo analogue **6**. Hydrolysis of **8** and subsequent methylation yields **5** as one biogenetic building block. The fact that **5** is devoid of activity in almost all studied cell lines suggests that at least two bromotyrosine derived units must be fused as present in **8** for obtaining appreciable activity in the studied cellular systems. Dimerization of a hemibastadin unit such as present in **8** which gives rise to bastadin derivatives (e.g., **1**–**3**) leads to a further enhancement of activity suggesting that the size of the molecule in addition to bromination of the aromatic rings is a further factor which influences the cellular activity of the studied compounds. 

### 2.2. Quantitative Videomicroscopy Analyses

**Figure 2:**
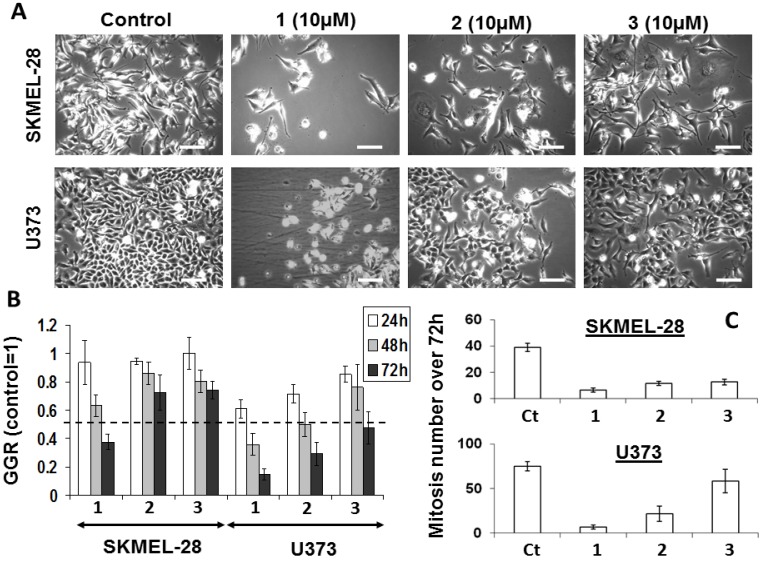
(**A**) Images of SKMLEL-28 and U373 cells left untreated or treated with 10 µM of bastadins at 10 µM during 72 h. Scale bar: 100 µm. (**B**) Global Growth Ratio for each experiment at 24 (open bars), 48 (gray bars) and 72 h (black bars) of treatment. (**C**) Number of mitoses that occurred over the 72 h period of observation for each experimental condition (control *versus* bastadin treated cells at 10 µM). Data are expressed as the means ± SEM.

While effective at determining the metabolic activity of cells, the colorimetric MTT assay cannot provide information as whether a compound decreases the global growth of normal or cancerous cells through cytotoxic, cytostatic, or anti-adhesive features or a mix of several of these features. To elucidate the specific features of bastadin derived anti-cancer effects, we used quantitative videomicroscopy to characterize these growth inhibitory effects [30,39]. The effects induced by bastadins-6, -9 and -16 (compounds **1**–**3**) on human SKMEL-28 melanoma and U373 glioblastoma cells are shown in Figure 2. We have chosen the concentration of 10 µM in order to compare the three cyclic bastadin effects on both cell lines at a similar dosis which corresponds to the IC_50_ of bastadin-16 on U373 cancer cells (11 µM) and which is closed to the IC_50_ values of bastadin-16 on SKMEL-28 (7 µM) and of bastadin-9 on both U373 and SKMEL-28 cells (7 µM).

The white and bright objects in Figure 2A correspond either to dying cells (e.g., cytotoxic effects) or to cells blocked in mitosis (cytostatic effects). A global growth ratio (the GGR index) was thus calculated for each compound (Figure 2). For both the controls and the treated cells, the global growth (GG) was first calculated by dividing the number of cells on the image at 24, 48 and 72 h by the number of cells on the first image. The GGR index was calculated by dividing the GG values calculated for the treated SKMEL-28 or U373 cancer cells by the GG values calculated for the control. As shown in Figure 2, at the same dosage of 10 µM, bastadin-6 (**1**) is the most potent compound, followed by bastadin-9 (**2**) and then bastadin-16 (**3**) for both cell lines, consistent with the potency data obtained with the colorimetric MTT assay (Table 1). In contrast, bastadin-9 (**2**) and -16 (**3**) appeared less active in the SKMEL-28 melanoma cells as assayed with quantitative videomicroscopy than by the MTT colorimetric assay (Table 1; Figure 2). The marked decrease in the total number of mitoses over the 72 h period observed with these compounds (marked effects except with bastadin-16 on U373 glioblastoma cells; Figure 2C) suggested that they exert their anti-cancer activities through primary cytostatic effects. These later were distinguished from cytotoxic effects on the basis of the dynamic movies and particularly the timelines associated with the compound-induced effects: blocking of proliferation was first observed and led, when sustained, at some cell death at the end of the experiment depending on the compound and the cell line under consideration with bastadin-6 (**1**) being the most cytotoxic one. These data suggest that the bastadin-mediated effects could at least partly depend on the cell cycle kinetic characteristics of the cancer cells analyzed. To further examine these effects, flow cytometry analyses were performed, as detailed in the next section.

### 2.3. Cell Cycle Kinetics versus Pro-Apoptotic Features

**Figure 3:**
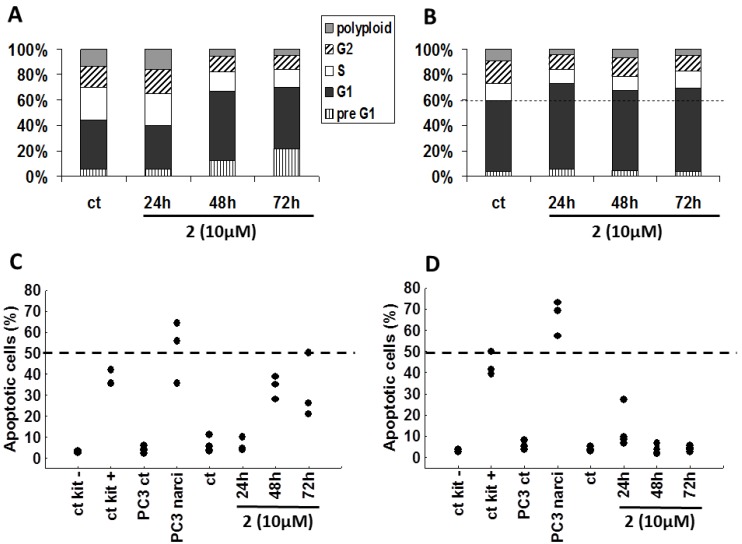
(**A** and **B**) Propidium iodide cell cycle analyses of SKMEL-28 and U373 cancer cells respectively, either left untreated or treated with 10 µM of (**2**). The results are presented as the means of the 3 replicates of the experiment. (**C** and **D**) Proportion of apoptotic cells (percentages) by TUNEL staining in SKMEL-28 and U373 cells, respectively. In addition to the negative and positive controls provided with the kit, we included two additional controls using PC-3 prostate cancer cells untreated or treated for 72 h with narciclasine (narci) at 1 µM. Each replicate result is presented as a black dot.

Using flow cytometry, we examined the influence of bastadin-9 (**2**, for which we had sufficient amount of material) on both the cell cycle kinetics and the apoptotic features in SKMEL-28 melanoma and U373 glioblastoma cells (Figure 3). Bastadin-9 (10 µM) decreased the proportion of proliferating SKMEL-28 melanoma cells characterized by a decrease in the S phase with an increase in G1 phase. This decrease is consistent with the cytostatic effects determined using quantitative videomicroscopy (Figure 2C). This compound increased in a time-dependent manner the proportion of dying cells in the preG1 fraction in SKMEL-28 melanoma cells only (Figure 3A as compared to Figure 3B for U373 cell line). TUNEL analyses determination of the levels of apoptotic cells confirmed the bastadin-9 (**2**) apoptosis induction in about one-third of the SKMEL-28 melanoma cells (Figure 3C), as also observed in the quantitative videomicroscopy experiments while no effects could be observed with respect to U373 glioblastoma cells (Figure 3B,D). In addition to the negative and positive controls furnished by the manufacturer, we also used a positive control, narciclasine (1 µM), which is an isocarbostyril compound isolated from *Narcissus* bulbs, on the PC-3 prostate cancer cells [36]. The pro-apoptotic effects induced by bastadin-9 (**2**) in SKMEL-28 melanoma cells (and subsequent cell death measured by increasing preG1 cells; Figure 3A) were of lower magnitude than those induced by narciclasine in PC-3 prostate cancer cells (Figure 3C). Nevertheless, **2** induced both cytostatic and then cytotoxic effects in SKMEL-28 melanoma cells, a feature that should correspond with several distinct mechanisms of action for exerting its growth inhibitory effects in these melanoma cells.

By contrast, it seems that bastadin-9 could overcome the intrinsic resistance of the U373 glioblastoma cells through pure cytostatic but not cytotoxic effects. The underlying molecular pathways have yet to be identified. As mentioned earlier, bastadins and congeners display activity with respect to the ryanodine-sensitive Ca^2+^ channels (ryanodine receptors; RyRs) [13,14,15]. Bastadin-induced cytotoxic and/or cytostatic effects could possibly be due, at least partly, to bastadin-induced modifications in the actin cytoskeleton organization through modulation of the RyRs [40,41], with modifications in the actin cytoskeleton organization leading to cytotoxic, not cytostatic, effects in endothelial cells [42] as well as in cancer [29,30,34] cells. As emphasized by Mackrill [43], RyRs are high conductance intracellular cation channels that release calcium ions from stores, such as the endoplasmic and sarcoplasmic reticulum, with altered RyR gating being implicated in a wide range of diseases, including cancer. However, Mackrill [43] also emphasizes that the available pharmacological tools for manipulating RyR gating are generally unsuitable for clinical, veterinary or agricultural use, owing to their lack of selectivity, their inappropriate solubility in the aqueous or lipid environment, or the generation of side-effects. It is possible that the bastadin-mediated cytostatic and/or cytotoxic effects in cancer cells occur by targeting the RyRs. Whether bastadins affect cells in a cytostatic and/or cytotoxic manner may depend on the type of cell studied and the concentrations used. 

While naturally occurring bastadins are difficult to isolate or to synthesize in large quantities, DBHB (**8**) can be easily synthesized in multigram amounts, supporting our decision to pursue our investigations with DBHB (**8**). Indeed, DBHB (**8**) turned out to exert cytostatic effects at its IC_50_ growth inhibitory concentration on B16F10 apoptosis-sensitive melanoma cells with marked cell shape modification (data not shown) while it behaves as an anti-migratory compound at lower concentrations devoid of any cytotoxic and/or cytostatic effects as illustrated in the next sections. Whether DBHB does or not target RyRs at these low nontoxic concentrations for which it displays anti-migratory effects remains to be determined. 

### 2.4. DBHB (8) Exhibits Anti-Angiogenic Activity

**Figure 4:**
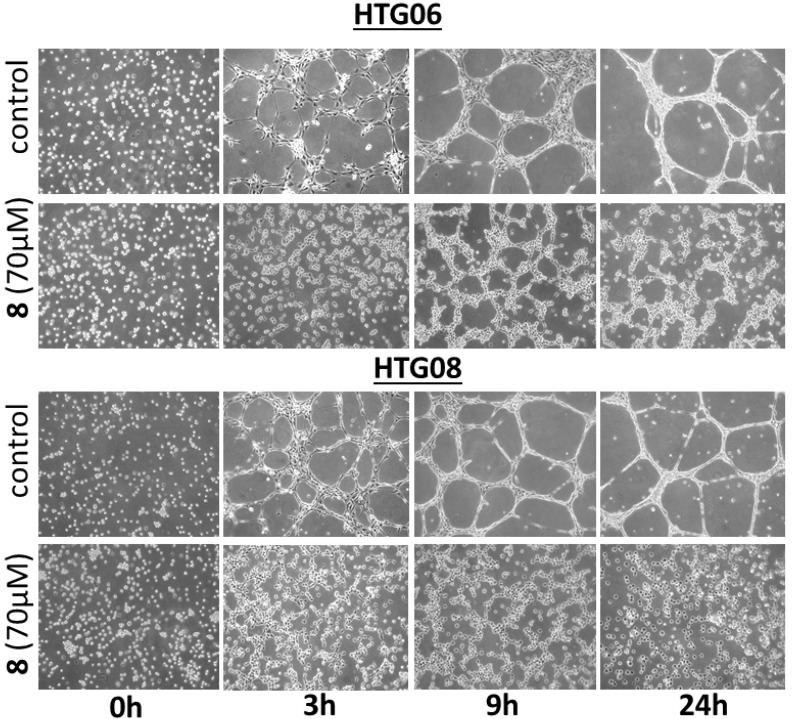
Representative images of the tubular network formation of HUVEC endothelial cells when cultured on Matrigel over a 24 h period. Two distinct HUVECs primary cultures, HTG06 and HTG08, were used. Treatment with DBHB (**8**) at 70 µM completely impaired the ability of endothelial cells to form these networks.

Guided by the fact that several naturally occurring bastadins are reported to exhibit anti-angiogenic effects [18,19,20,21], we evaluated if DBHB (**8**) also exhibits anti-angiogenic effects. Treatment of two human umbilical vein endothelial cell (HUVEC) lines, *i.e.*, HTG06 and HTG08, with 70 µM DBHB (**8**; the mean IC_50_ concentration on cancer cells; Table 1) induced marked anti-angiogenic effects in both HUVEC lines, as illustrated in Figure 4. The DBHB-induced inhibition of the HUVEC tubulogenesis correlated with anti-migratory but not cytotoxic effects, as shown in Figure 4. As shown below, DBHB (**8**) also exhibits anti-migratory effects without cytotoxic and/or cytostatic effects in melanoma cells at even lower concentrations than those used here for analyzing DBHB-induced anti-angiogenic effects.

Aoki *et al.* [21] observed that bastadin-6 inhibits vascular endothelial growth factor (VEGF)- or basic fibroblast growth factor (bFGF)-dependent proliferation of HUVECs with a 20- to 100-fold selectivity in comparison with normal fibroblasts (3Y1) or several tumor cell lines (KB3-1, K562 and Neuro2A). These authors also reported that bastadin-6 inhibited the VEGF- or bFGF-induced tubular formation, the VEGF-induced migration of HUVECs and the VEGF- or bFGF-induced *in vivo* neovascularization in the mice corneal assay. Bastadin-6 also suppressed the growth of s.c. inoculated A431 solid tumor in immunodeficient mice at 100 mg/kg (intraperitoneal) [21]. While bastadin-6 has been reported to induce apoptotic cell death in HUVECs [21], our data on DBHB (**8**) reported here, suggest a mechanism of action that is anti-migratory but non-cytostatic and non-cytotoxic in HUVECs: when considering evolution of HUVEC cell populations over the first hours, we observed marked anti-angiogenic effects while cellular spreading and cell population evolution are still present (Figure 4). Our observations with DBHB (**8**) in endothelial cells could therefore differ from those reported by Aoki *et al.* [21] for bastadin-6. 

### 2.5. Direct Impact of Serum on the Anti-Migratory Properties of DBHB (**8**) in B16F10 Melanoma Cells, with a Potential Involvement of Albumin

The anti-migratory effects observed above with endothelial cells led us to investigate whether DBHB could display anti-migratory effects on cancer cells. We made use of quantitative scratch wound assays [29,30] for this purpose with the mouse B16F10 melanoma cells.

**Figure 5:**
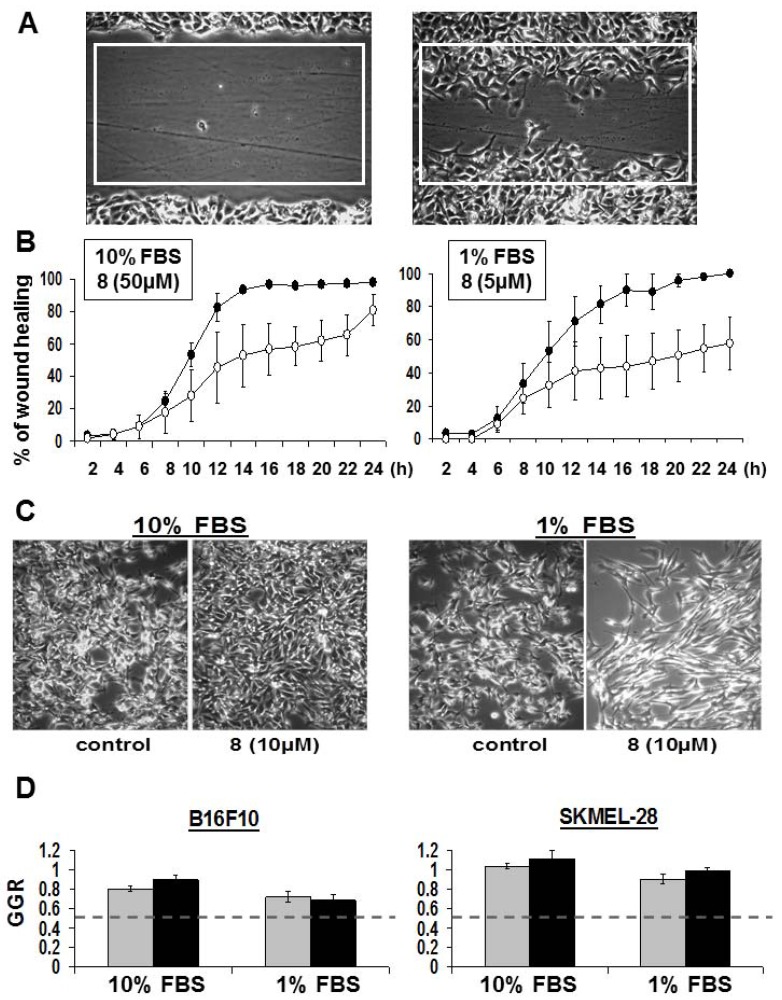
Anti-migratory effects of DBHB (**8**) on cancer cells. (**A**) Image of a wound made in confluent B16F10 cells at t = 0 h and 6 h after. Quantification of the area of the white rectangle healed over time is shown in (**B**) for cells cultured in 10 *vs.* 1% FBS. Black dots: control; open dots: DBHB treated condition at 50 µM or 5 µM as indicated. (**C**) Images of B16F10 cells left untreated or treated for 48 h with DBHB at 10 µM. (**D**) Quantitative data of B16F10 and SKMEl-28 cell growth ratio (control condition GGR = 1) when treated with 10 µM of DBHB for 24 h (gray bars) and 48 h (black bars). All data are expressed as the mean ± SEM.

An image of a B16F10 melanoma scratch wound at “0 h” and the same scratch wound after 6 h of culture are shown in Figure 5A. The area percentages covered by B16F10 melanoma cells colonizing the white rectangle in the scratch wound over time have been quantitatively determined by means of computer-assisted phase-contrast microscopy [29,30]. The left panel of Figure 5B clearly shows that DBHB (**8**) only delayed the wound healing process of B16F10 mouse melanoma cells. To identify whether this effect related to anti-migratory and/ or to anti-proliferative effects, we decreased serum concentration to 1% to minimize the proliferative contribution to the wound healing. While not expected, we discovered that the 50 µM DBHB concentration when used in 1% FBS culture medium led to very high cytotoxic effects with cell death occurring within the first two hours of treatment (data not shown). We had to decrease DBHB till 10 µM to avoid any effect on cell proliferation as assessed by quantitative videomicroscopy in Figure 5C,D. Similar data were obtained with respect to SKMEL-28 cells (Figure 5D). Interestingly, the B16F10 cell shape morphology seemed more affected by the DBHB in a 1% FBS culture than at the same concentration of DBHB in 10% FBS, a feature that could, at least partly, explain the anti-migratory effects observed (Figure 5B).

We thus compared the recolonization of the wound induced by 50 µM DBHB (*i.e.*, the IC_50_ growth inhibitory concentration of DBHB as measured using the MTT colorimetric assay (Table 1)) when B16F10 melanoma cells were cultured in medium supplemented with 10% of FBS to the ones induced by 5 µM in 1% FBS supplemented medium. The data in Figure 5B show that the 50 µM DBHB delayed the B16F10 melanoma cell migration during the first 24 h of observation and then this anti-migratory effect was lost. Lowering the DBHB concentration from 50 to 5 µM and the serum concentration from 10 to 1% induced higher DBHB anti-migratory effects than in the previous experimental conditions. At these lower DBHB and serum concentrations, a 50% inhibition of the B16F10 migration was still observed at 24 h when full colonization (100%) of the rectangle had been completed by the B16F10 melanoma cells in the control condition (Figure 5B). Thus, while lowering the FCS concentration from 10 to 1% only slightly delayed but did not impair the B16F10 cell migratory properties (see the control curves in Figure 5B), this ten-fold decrease in serum concentration allowed the even reduced DBHB concentration to inhibit cell migration more markedly.

The anti-migratory (non-cytostatic and non-cytotoxic) effects of DBHB in endothelial (Figure 4) and cancer cells (Figure 5A,B), are consistent with previously published data on bastadin- and DBHB-related antifouling properties [11,12]. Indeed, DBHB induced an inhibition of the larval settlement of *Balanus improvisus* without being toxic to either these larvae or the brine shrimp larvae [12]. By contrast, cyclic bastadins are toxic with poor, if any, antifouling activity at nontoxic concentrations [12].

**Figure 6:**
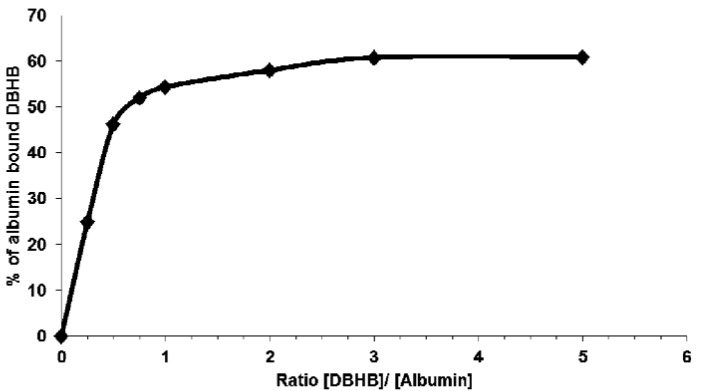
Binding curve of DBHB to albumin. Indirect quantification of bound DBHB (**8**) was performed after TCA precipitation. Data are presented for ratio of DBHB/ albumin of 1/4 to 5/1.

Considering that the serum concentration affected the DBHB-induced effects on cell growth, death and migration (Figure 5), we hypothesized that DBHB could interact with albumin, one of the major components of plasma. As shown in Figure 6, DBHB binds strongly to albumin, whose concentration could thus impair DBHB-induced anti-migratory effects on both cancer and endothelial cells *in vivo*. 

### 2.6. *In Vivo* Analyses of DBHB (**8**) Activity in B16F10 Melanoma-Bearing Mice

The *in vitro* anticancer activity in 6/6 cancer cell lines (Table 1) and the anti-angiogenic effects in 2/2 HUVEC lines of DBHB led us to assay this compound’s *in vivo* anticancer activity. The formulation we developed for DBHB to deliver 40 mg/kg with chronic i.v. administrations of the compound (*i.e.*, 200 µL of i.v. administration volume at a concentration of 5 mg/mL) to 25 g mice was made in NaCl 0.9% (see Experimental for details). The formulation was a relatively homogeneous suspension of DBHB compatible with chronic i.v. administrations (data not shown).

Preliminary toxicological experiments revealed that chronic i.v. administrations of 40 mg/kg DBHB to healthy mice (not grafted with tumors) induced no toxic side effects in terms of mouse behavior, weight and survival (data not shown). This dosage could lead in theory to a 10 fold IC_50_ plasmatic concentration at the injection time.

We then analyzed the DBHB *in vivo* antitumor activity using the mouse B16F10 melanoma model that, once injected i.v. in the tail vein of mice, rapidly develop aggressive lung pseudometastases [44]. The non-toxic dose of 40 mg/kg dose of DBHB (**8**) was chronically injected i.v. nine times, *i.e.*, three times a week (Monday, Wednesday, Friday) for three consecutive weeks, with the first injection occurring on the 5th day post-tumor cell grafting to mice bearing B16F10 melanoma-related lung pseudometastases. On the 5th day post B16F10 melanoma cell injection in the tail, lung pseudometastases can already be found at the histological level. Temozolomide was used as a reference compound and administered at the same dose (40 mg/kg) as DBHB and schedule, as we previously reported for this compound with the B16F10 melanoma model [44].

At these conditions, DBHB had no significant *in vivo* therapeutic benefits in the B16F10 model, neither as a single agent nor in combination with temozolomide (data not shown). To fully evaluate DBHB for *in vivo* anticancer activity, the development of specific formulations to prevent the albumin binding in the plasma is needed for the future.

## 3. Experimental

### 3.1. Sample Collection, Extraction and Purification of bastadin 6 (**1**), bastadin 9 (**2**) and bastadin 16 (**3**)

A specimen of the marine sponge *Ianthella basta* was collected at Ambon Tanjung Island, Indonesia in August 1996 and stored in ethanol at −20 °C until extraction. Taxonomic identification was performed by the Zoological Museum, Amsterdam, The Netherlands (reference number ZMAPOR17857). After freeze drying, the material (121 g) was ground and then macerated with acetone, followed by methanol. Each extraction cycle was performed four times for five hours with 800 mL of solvent. Acetone and MeOH extracts were combined and evaporated under vacuum to yield a dried crude extract of approximately 30 g in weight. A liquid-liquid partition led to four fractions (hexane 2 g; EtOAc 5 g; *n*-BuOH 3.5 g; H_2_O 17.1 g). HPLC-DAD analysis indicated the EtOAc- and BuOH fraction to be of further interest. The EtOAc fraction was further subjected to vacuum liquid chomatography (VLC) using a gradient system from hexane over EtOAc over CH_2_Cl_2_ to MeOH to obtain 21 fractions. Compound **1** was eluted with 45% hexane/55% EtOAc, **2** with 40% hexane/60% EtOAc and both were further purified by size exclusion chromatography in 100% MeOH and semi-preparative HPLC utilizing an appropriate gradient system to yield 21.6 mg (**1**) and 1.9 mg (**2**), respectively. Bastadin 16 (**3**, 34.2 mg) were obtained from the BuOH fraction after further purification via size exclusion chromatography in 100% MeOH and semi-preparative HPLC.

### 3.2. Chemical Syntheses

*Methyl-[2-hydroxyimino-3-(4-hydroxyphenyl)]-propionate* (**4**). 4-Hydroxyphenylpyruvic acid (3 mmol) was suspended in H_2_O (50 mL) and NaOH (5%) was added to dissolve the acid completely. Hydroxylamine·HCl (20 mmol), dissolved in H_2_O (20 mL) was added to the solution and the pH was adjusted to approx. 8–9 with 5% NaOH. This solution was stirred for one hour at 60 °C and thereafter the pH was readjusted to 8–9 with 5% NaOH. The solution was heated once more for two hours at 60 °C and was left to cool down to room temperature. The cold solution was adjusted with HCl to pH 1 and extracted with ether. The obtained residue was recrystallized from ether/petroleum ether (60–80 °C) to yield [2-hydroxyimino-3-(4-hydroxyphenyl)] propionic acid as white crystals. The oxime (4 mmol) was dissolved in dimethylformamide (30 mL) and diazabicycloundecane (DBU, 4 mmol) was added. After cooling on ice methyl iodide (20 mmol) was added and the reaction was left stirring at 4 °C for 3 h. Thereafter water (100 mL) was added and the solution was extracted with ether. The traces of dimethylformamide were removed from the ether residue under vacuum. The product was recrystallized from ether/petroleum ether (60–80 °C) to yield white crystals of **4**, which served also as a precursor for the following syntheses of **5**, **6** and **8**.

*Methyl-[2-hydroxyimino-3-(3,5-dibromo-4-hydroxyphenyl)]-propionate* (**5**). The dibromo methylester **5** was prepared by a bromination of **4**. A bromine solution (0.1 M) was freshly prepared in dichloromethane and 50 mL of this solution were added to an ether solution of **4** (1 mmol). The reaction was left for 24 hours at room temperature. The organic solution was washed with diluted sodium hydrogen sulphite and sodium hydrogen carbonate solution and dried under reduced pressure. The residue was purified by chromatography on silica gel to give the main product of **5**.

*Norbromohemibastadin-1* (**6**). Norbromohemibastadin-1 (**6**) was synthesized as reported earlier [12].

*l-Tyrosine-tyramide A* (**7**). l-Tyrosine-tyramide A (**7**) was synthesized as reported earlier [12].

*5,5'-Dibromohemibastadin-1* (**8**). DBHB (**8**) was synthesized as reported earlier [12].

### 3.3. Determination of the *In Vitro* Growth Inhibitory Concentrations

Six cancer cell lines were obtained from the European Collection of Cell Cultures (ECACC; Salisbury, UK), the American Type Culture Collection (ATCC; Manassas, VA, USA) or the Deutsche Sammlung von Mikroorganismen and Zellkulturen (DSMZ, Braunschweig, Germany). These six cell lines included the MCF-7 breast cancer (DSMZ code ACC115), the A549 NSCLC (DSMZ code ACC107), the Hs683 oligodendroglioma (ATCC code HTB-138), the U373 glioblastoma (ECACC code 89081403), and the SKMEL-28 (ATCC code HTB-72) and B16F10 (ATCC code CRL-6475) melanoma cell lines. The cells were cultured in RPMI (Lonza, Verviers, Belgium) medium supplemented with 10% heat inactivated fetal bovine serum (Lonza). All culture media were supplemented with 4 mM glutamine, 100 µg/mL gentamicin, and 200 U/mL penicillin and 200 µg/mL streptomycin (Lonza). The overall growth level of the human cancer cell lines was determined using a colorimetric MTT (3-[4,5-dimethylthiazol-2-yl-diphenyltetrazolium bromide, Sigma, Diegemy, Belgium) assay as detailed previously [34,35,36]. Each experimental condition was performed in six replicates.

### 3.4. Computer-Assisted Phase Contrast Microscopy (Quantitative Videomicroscopy)

The direct visualization of the compound-induced cytostatic and/ or cytotoxic effects for the human U373 glioblastoma, human SKMEL-28 melanoma and mouse B16F10 melanoma cells was recorded as described previously [30,39]. Briefly, the quantitative videomicroscopy experiment was designed to capture digital images of the cell culture every four minutes for a 72 h period, providing 1,080 digitized images that can be visualized as dynamic movies that were approximately 1 min in length [30,39]. 

### 3.5. Flow Cytometry

Cell cycle analysis (propidium iodide staining) and apoptosis detection were performed simultaneously in U373 and SKMEL-28 cells with the APO TUNEL detection kit (BD Pharmingen, Erembodegem, Belgium) following the manufacturer’s recommendations. A similar procedure was described in [44]. Narciclasine, an isocarbostyril isolated from *Narcissus* bulbs was assayed on PC-3 prostate cancer cells (DSMZ code ACC465) as a positive control for the apoptosis measurements [36]. The experiment was performed once in triplicate.

### 3.6. *In Vitro* Anti-Angiogenesis Analyses

Human HUVECs were established as primo cultures according to a method we described previously [42]. Their ability to form tubular networks was evaluated by seeding 100,000 cells/well in a six well plate containing pure Matrigel (BD Pharmingen) [42]. Experiments were conducted once, in triplicate, with 5 images per well taken at time = 0 h, 3 h, 6 h, 9 h and 24 h.

### 3.7. Formulating DBHB (8) for *in Vivo* Analyses

DBHB was formulated as a suspension at a concentration of 5 mg/mL in a solution of NaCl 0.9% for i.v. infusion (B. Braun, Diegem, Belgium). To ensure homogeneity, the pre-mix was vortexed and then homogenized using a high speed homogenizer composed of an IKA® T10 Basic rotor connected to an SN10 G5 dispersing element (Boutersem, Belgium) at a speed of 24,000 rpm for 5 min in an ice bath to avoid temperature increases during the process, which could possibly damage the compound. A solution of NaCl 0.9% for infusion was chosen as the dispersant medium to ensure that the injection suspension has the same osmolality as the blood fluid. 

### 3.8. The *in Vivo* Model of Lung Pseudometastases from Mouse B16F10 Melanoma

We injected 250,000 B16F10 melanoma cells per mouse into the tail vein of C57Bl/6 6 week old female mice (Charles River, France). Lung pseudometastases developed, leading to animal death within 3 to 4 weeks without treatment [44]. Eleven mice per experimental condition were used. Treatments used in the present study are detailed in section 3.6 of Results and Discussion. The experiment was conducted with the authorization no. LA1230568 of the Animal Ethics Committee of the Federal Department of Health, Nutritional Safety, and the Environment (Belgium). 

### 3.9. Scratch Wound Assay

The procedure used in the scratch wound assays was described previously [29,30]. Briefly, cells were seeded and cultured in 25 cm² flasks with 10% FBS until confluence. The wound was manually performed with a 200 µL pipette tip. Cells were washed before exposure to compound **8** at the appropriate FBS concentration (1 or 10%) or left untreated in the same medium and placed in the videomicroscopy incubator device. The experiments were conducted once, in quadruplicate. We were unable to use the software for one sample because of contrast difficulty (n = 3 for the control condition at 1% FBS). 

### 3.10. Determination of DBHB (8) Affinity to Albumin

The albumin binding affinity of **8** was investigated with an HPLC based assay. A DBHB (**8**) stock solution (0.1 mM in 50% MeOH) was prepared and differing amounts of the stock were added to seven different 2 mL glass vials containing an albumin (Sigma-Aldrich) solution (0.1 mM in 50% MeOH). MeOH (50%) was added to end up with molar ratios (DBHB: albumin) of 1:4, 1:2, 3:4, 1:1, 2:1, 3:1 and 5:1 in a final volume of 2 mL. The samples were mixed thoroughly by utilizing a vortex for approximately 10 s each and subsequently incubated under shaking (200 rpm) at 37 °C for 1 h. Then, all samples where centrifuged at 13.300 rpm for 10 min. 50 µL of the supernatant of all samples were injected into a Dionex Ultimate 3000 HPLC System and the concentration was calculated on the basis of quantification via AUC integration. A three point calibration was performed with DBHB (**8**) samples, treated identically to the samples above. The amount of DBHB (**8**) was determined indirectly as follows:

n (bound) = n (100%) − n (free)

## 4. Conclusions

Bastadins, at least bastadin-6, -9 and -16 (compounds **1**–**3**), exhibit cytotoxic *versus* cytostatic effects at single digit µM concentrations for several mouse and cancer cell lines. While the anti-cancer effects are of similar levels in cancer cells sensitive to pro-apoptotic stimuli *versus* cancer cells displaying various levels of resistance to pro-apoptotic stimuli, the type of effects, *i.e.*, cytotoxic *versus* cytostatic, depend on the cell type analyzed. With an approximately ten times weaker *in vitro* growth inhibitory effect on the investigated cancer cell lines compared with bastadin-6, -9 and -16 (**1**–**3**), DBHB (5,5'-dibromohemibastadin; (8) exhibited both anti-angiogenic (HUVECs) and anti-migratory effects in mouse B16F10 melanoma cells. The anti-migratory effects that appeared at one-tenth of the IC_50_ *in vitro* growth inhibitory concentration were antagonized by increasing percentages of serum in the culture media of the B16F10 melanoma cells. Further experiments demonstrated that DBHB bound strongly to albumin, possibly explaining the treatment failure of DBHB delivered through the i.v. route in an *in vivo* tumor model. Anti-migratory compounds that decrease the migration levels of cancerous cells may increase sensitivity of those cancer cells to the cytotoxic damages experienced with conventional chemotherapy and radiotherapy. This suggests that an anti-migratory compound such as DBHB, which is also anti-angiogenic, could be delivered prior to conventional radiotherapy and/or chemotherapy to sensitize migrating cancer cells to these conventional therapies. 

While our *in vivo* experiments with DBHB failed to increase the survival of B16F10 melanoma-bearing mice, it may be possible in the future to develop inhalation formulations to deliver DBHB directly to the lung (thus avoiding albumin binding) as we recently demonstrated for temozolomide [45,46]. Lung cancer may be an ideal first clinical target for these compounds because it is a deadly disease with dismal prognoses and a high resistance to conventional as well as targeted therapies [47,48,49]. The local delivery of an anti-migratory, but non-cytostatic and non-cytotoxic, agent such as DBHB, with limited systemic side effects, could contribute added therapeutic benefits to conventional cytotoxic radiotherapy and chemotherapy and even targeted therapies (kinase inhibitors; anti-receptor antibodies) in the specific combat against lung cancers.
